# Progress in the diagnosis of lymph node metastasis in rectal cancer: a review

**DOI:** 10.3389/fonc.2023.1167289

**Published:** 2023-07-13

**Authors:** Wei Peng, Huimin Qiao, Linfeng Mo, You Guo

**Affiliations:** ^1^ Medical Big Data and Bioinformatics Research Centre, First Affiliated Hospital of Gannan Medical University, Ganzhou, Jiangxi, China; ^2^ School of Public Health and Health Management, Gannan Medical University, Ganzhou, Jiangxi, China; ^3^ School of Health and Medicine, Guangzhou Huashang Vocational College, Guangzhou, Guangdong, China

**Keywords:** rectal cancer, lymph node metastasis, diagnosis, OMICS data, imaging data

## Abstract

Historically, the chief focus of lymph node metastasis research has been molecular and clinical studies of a few essential pathways and genes. Recent years have seen a rapid accumulation of massive omics and imaging data catalyzed by the rapid development of advanced technologies. This rapid increase in data has driven improvements in the accuracy of diagnosis of lymph node metastasis, and its analysis further demands new methods and the opportunity to provide novel insights for basic research. In fact, the combination of omics data, imaging data, clinical medicine, and diagnostic methods has led to notable advances in our basic understanding and transformation of lymph node metastases in rectal cancer. Higher levels of integration will require a concerted effort among data scientists and clinicians. Herein, we review the current state and future challenges to advance the diagnosis of lymph node metastases in rectal cancer.

## 1 Introduction

Lymph node metastasis is complex and its progression involves diverse processes in the patient’s body. Consequently, the cancer research community has generated massive omics and imaging data to study the hallmarks of cancer as comprehensively as possible. The fast accumulation of massive omics and imaging data catalyzed by the rapid development of advanced technologies has driven improvements in the accuracy of diagnosis of lymph node metastasis. The accurate detection of lymph node metastasis and staging is essential and well-acknowledged for making appropriate treatment plans and prognostic predictions for these patients (1). However, clinical staging is subjective and influenced by many factors, an important reason for the high rate of missed diagnosis. Therefore, it is necessary to seek more effective detection methods for identifying lymph node metastasis.

The diagnosis of lymph node metastasis plays an essential role in the field of rectal cancer. First, lymph node metastasis is the main mode of metastasis which occurs in 30–40% of patients with rectal cancer, and the local recurrence rate is very high, which is also closely related to their prognoses (2–4). Second, the treatment of rectal cancer depends on the stage (5). The TNM staging system is the basis of treatment formulation and facilitates the assessment of the prognoses of patients with rectal cancer by physicians (6). Moreover, preoperative evaluation of lymph node metastasis can provide critical information for determining the necessity of neoadjuvant therapy and the adequacy of surgical resection (7–9). However, the current clinical accuracy of N-stage remains unsatisfactory (10).

Lymph node metastasis in rectal cancer has immense scope and it is nearly impossible to cover everything in one review. Therefore, herein, we focus on key diagnostic analyses that have led to conceptual advances in our understanding of cancer biology and impacted decision-making for disease treatment. Further, we detail the reviews in pertaining sections to direct interested readers to relevant resources. We acknowledge that our limited selection of topics and examples may omit important work, for which we sincerely apologize.

**Figure 1:**
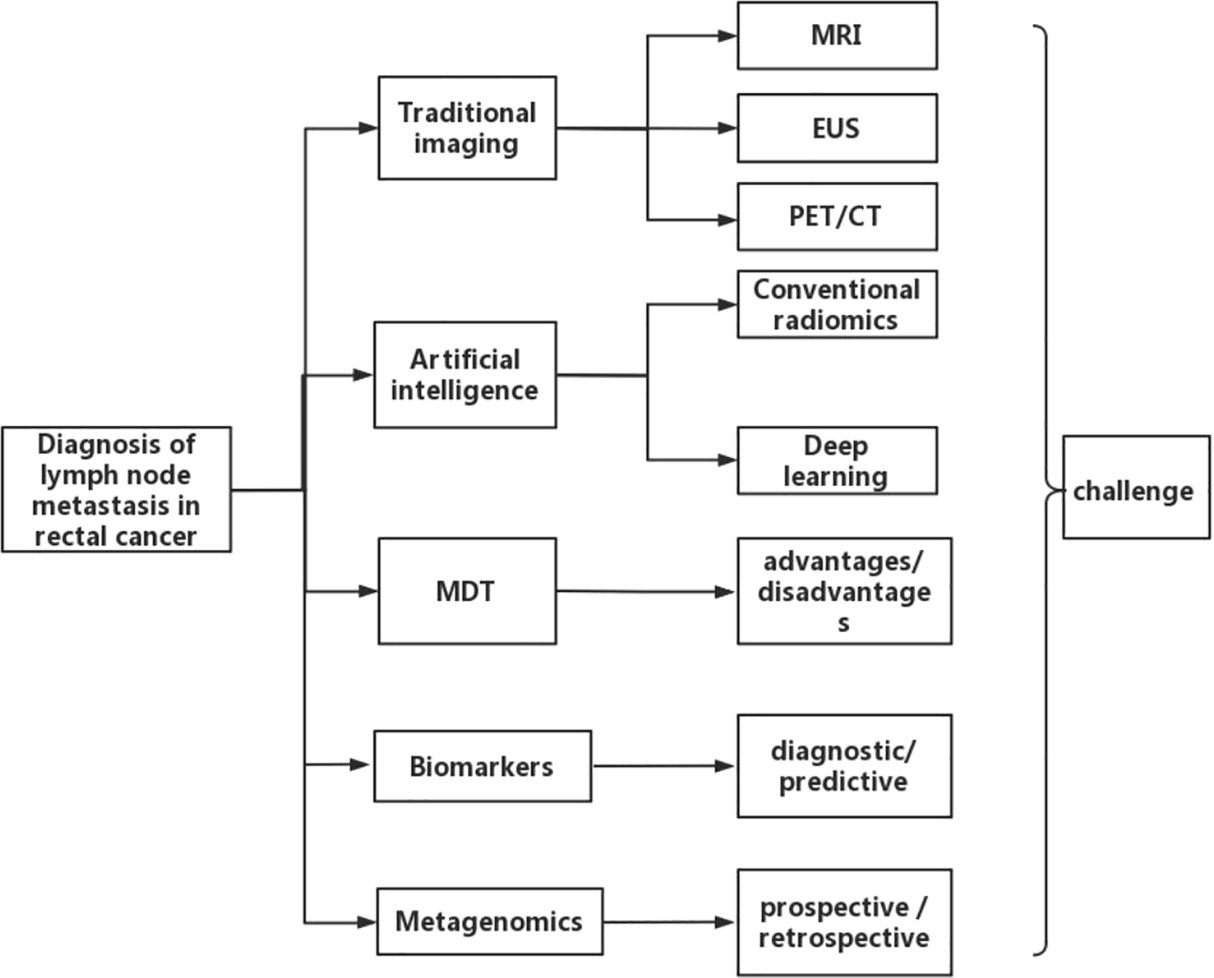
The content framework of the entire article.

In this review, studies were systematically searched in PubMed before December, 2022. The search terms used were ((rectal cancer [MeSH Terms]) AND (diagnosis [MeSH Terms]) AND (lymph node metastasis [MeSH Terms])). The “Similar articles” function was used to broaden the search, and all citations were considered for relevance. A manual search of the references of publications was carried out to ensure that no relevant studies were excluded. We begin by describing conventional imaging examination, including magnetic resonance imaging (MRI), endoscopic ultrasonography (EUS), and positron emission tomography/computed tomography (PET/CT) for the diagnosis of N staging of rectal cancer. Different imaging methods vary in the range of their diagnostic accuracy and their benefits and drawbacks. These examination techniques have certain limitations in determining lymph node metastasis in rectal cancer. Presently, artificial intelligence, multidisciplinary team (MDT), biomarkers, and metagenomics methods have simultaneously increased the accuracy of determining lymph node metastasis in rectal cancer. Finally, we discuss current challenges and the future direction of the field. The overall framework of this paper is shown in **Figure 1**. This review consists solely of a succinct assessment of the development in research on the diagnosis of lymph node metastases in rectal cancer.

## 2 Traditional imaging examination-based assessment of lymph node metastases in rectal cancer

### 2.1 MRI

MRI plays a major role in assessing the staging of rectal cancer. It can indicate the precise anatomy of the rectum and mesenteric fascia, and accurately predict the margin of circumferential resection and tumor staging. The guidelines of the European Society of Gastrointestinal and Abdominal Radiology recommend MRI for diagnosing the staging of rectal cancer (11). Its better spatial resolution is advantageous in distinguishing benign from malignant lymph nodes. In the early stage of diagnosis of lymph nodes, their size is used as a diagnostic criterion but the utility of the boundary value of lymph node diameter remains inconclusive. Due to the lack of unified standards, the accuracy of determining the nature of tumor lymph nodes is not ideal, and different studies have the problem of over- or under-staging (12–14). MRI cannot completely determine the status of lymph nodes and the criteria used to determine lymph node metastasis may be different in different institutions (15). The accuracy of the results of MRI to detect lymph node metastasis is shown in **Table 1**. As shown, the results of different studies vary greatly and are not very accurate overall. Subsequently, the criteria of MRI for the detection of lymph node status should be unified to assist in accurate and individualized treatment of rectal cancer.

**Table 1:** Accuracy of the results of MRI for detecting lymph node metastasis.

PN/LN	Design	Sensitivity (%)	Specificity (%)	PPV (%)	NPV (%)	Accuracy (%)	Reference
354 120 324 58 30 90 25 21 217 56 22 109 60 87 148 327 205LN 126LN 257LN	P P R R R P R P R P P R R P R R P P P	82.8 64.60 47.5 52 94 71 85 66.7 85 75 71 79 72.0 77.50 70.40 45.6 58.0 65 80	58.1 51.40 77.3 77 67 86 69 75.0 41 67.30 70 37 45.7 74.50 55.90 84.8 88.4 75 98	62.8 47.00 45.3 - 81 83 58 66.7 58 60 63 76.5 - 72.10 - - 61.7 19 -	79.9 68.50 78.8 - 89 75 90 66.7 60 86.10 78 39 - 79.60 - - 86.7 96 -	69.5 56.70 - 66 83 79 74 71.4 63 82.10 - 67 56.7 75.90 61.30 78.0 76.1 - 95	(16) (17) (18) (19) (20) (21) (22) (23) (24) (25) (26) (27) (28) (29) (30) (31) (32) (33) (34)

PN, patient number; LN, lymph nodes; P, prospective; R, retrospective; PPV, positive predictive value; NPV, negative predictive value.

### 2.2 EUS

EUS examination is also a method commonly used for patients with rectal cancer. For the staging of superficial tumors, EUS is preferred (35). As the depth of invasion increases, the penetration of ultrasound to the tumor decreases, thereby decreasing the diagnostic accuracy. A systematic study (36) showed that the sensitivity and specificity of EUS in the diagnosis of lymph node involvement were approximately 0.81 (95%CI, 0.71-0.89) and 0.88 (95%CI, 0.80-0.94), respectively. A meta-analysis (37) for evaluating lymph node metastasis included data from 123 studies that showed that the sensitivity and specificity of EUS were 0.57 and 0.80, respectively. However, its ability to detect lymph nodes is limited by the difficulty in detecting smaller nodes or those closest to the tumor. Tombazzi CR and colleagues (38) have shown that EUS is very accurate for tumor staging of early rectal cancer. However, in fact, as it is difficult to distinguish between inflammatory and metastatic lymph nodes, the judgment of lymph node metastasis by EUS is not accurate, leading to misdiagnosis and possible overtreatment. The scope of EUS is also restricted, which excludes complete exploration of the pelvic cavity or iliac fossa. These limitations weaken its effectiveness in evaluating the staging of lymph nodes. In summary, EUS is not an ideal method for diagnosing lymph node metastasis in rectal cancer.

### 2.3 PET/CT

PET/CT imaging allows better observation of the health of the entire human body and the study of its anatomical structure and physiological function. It is often used in the clinical detection of early tumors, metastatic lesions, and lymph nodes qualitatively. 18F-FDG PET/CT is the most commonly used PET/CT imaging technique, which is a non-invasive examination method that combines morphological and functional information to diagnose tumors, perform differential diagnosis, assess clinical staging, curative effect monitoring, and determination of radiotherapy plan, bearing great significance (39, 40). 18F-FDG PET/CT imaging is an important imaging tool to evaluate patients’ conditions before surgery (41). This imaging technology can be utilized to assess the N staging of rectal cancer and provide accurate clinical staging and preoperative evaluation (42). A previous study (43) showed that when the cut-off value of the maximum standardized uptake of the lateral pelvic lymph nodes was set to 1.5, it was reasonable in predicting the risk of lateral pelvic lymph node metastasis in patients with rectal cancer. The sensitivity, specificity, positive predictive value, negative predictive value, and false negative value reached 82%, 93%, 58%, 98%, and 18%, respectively. Although the PET/CT technique can provide an accurate diagnosis of lymph node metastases in rectal cancer, it has certain limitations and cannot fully reflect the internal structure of lymph nodes, making accurate diagnoses difficult (44). Further, PET findings of lymph nodes with high FDG uptake levels may indicate that lymph nodes have metastasized, however, this condition is often misidentified as lymph node metastasis due to certain vascular structures (including venous femoral veins or inflammatory lymph nodes) (45). Taken together, future research should focus on improving the display of the internal structure of the lymph nodes, combined with the consequences of PET/CT to improve the accuracy of the diagnosis of lymph node metastasis in rectal cancer.

## 3 Artificial intelligence to evaluate lymph node metastases in rectal cancer

### 3.1 Conventional radiomics

The conventional radiomics workflow is typically based on extracting meaningful information from an area being studied. In recent years, radiomics has been used to evaluate several tumors and is increasingly being applied clinically to improve the accuracy of diagnosis, prognosis, and prediction of cancer. The results of the research of Huang YQ et al. (46) showed that the accuracy of detecting lymph node metastasis in rectal cancer could be improved by constructing a radiomic nomogram with radiomic characteristics, CT-reported lymph node status, and clinical risk factors. The distinguishing ability, evidenced by the area under the curve (AUC) was 0.778 (95%CI, 0.769-0.787), indicative of a good practical application value. Similarly, a retrospective study (47) showed that features of radiomics combined with a nomogram comprising a random score of 3, age, and lymph node size showed good discrimination, and the AUC value reached 0.884, suggestive of its high accuracy in the prediction of preoperative lymph node metastasis in patients with rectal cancer. Recently, Chen LD et al. (48) developed a multi-parameter nomogram, which could more accurately predict lymph node metastasis. This model organically integrated CT and S-wave elastic imaging techniques and had a high C-index of 0.857 (95% CI, 0.726–0.989). All the above studies showed good predictive efficacy for metastatic lymph nodes of rectal cancer but the models are different owing to the equipment type and parameters, making it difficult to evaluate an optimal model. The clinical application and promotion of imaging big data are needed to establish unified standards and multi-center data support.

### 3.2 Deep learning

The application of computer technology to the medical field has been a major breakthrough, allowing clinicians to make more accurate diagnoses and treatment plans. With the application of computer technology in the diagnosis of lymph node metastasis, the incidence of rectal cancer has reduced significantly in China and greatly improved the accuracy of its diagnosis, treatment, evaluation, and prediction (49). Because it is difficult to obtain massive data from medical images sometimes, transfer learning can be used. Transfer learning is a type of deep learning that uses pre-trained models and requires fewer medical images. This method first uses pre-trained weights from similar architecture networks to initialize the network, and then fine-tunes the parameters to suit the target application, with the last fully connected layer usually replaced by neurons of a new class based on the number of classes in the new classification task. Ichimasa K et al. (50) retrospectively analyzed the data of 690 consecutive T1 colorectal cancer patients and established intelligent model Mo1724, wherein 45 clinicopathological factors were analyzed to predict positive or negative lymph node metastases. The results suggested that the sensitivity of the model was 100% (95% CI, 72%–100%), the specificity was 66% (95% CI, 56%–76%), and the accuracy rate was 69% (95%CI, 59%–78%). Compared to the current guidelines, the AI model significantly reduces the number of missed cases of positive lymph node metastases. On this basis, Kudo SE et al. (51) used an artificial neural network to conduct in-depth studies on patients with lymph node metastasis according to their age, sex, tumor size, location, morphology, lymphatic and vascular infiltration, and histological grade. The results showed that in the verification cohort, the AUC value of patients with lymph node metastasis identified by the model was as high as 0.83, while that of patients with lymph node metastasis identified by following the guidelines was only 0.73 (P<0.001). After analysis, limited to patients initially undergoing endoscopic resection, the model showed that the AUC for patients with lymph node metastasis was still as high as 0.84, while the corresponding value with the guidelines was 0.77 (P=0.005). The model can be used to identify the need for additional lymph node dissection after endoscopic resection in patients with T1 colorectal cancer. The development of artificial intelligence technology has a useful guiding role in the diagnosis, treatment, and prognosis of patients with rectal cancer lymph node metastasis. Li J (52) used deep transfer learning to classify the lymph node status of patients with rectal cancer in an attempt to improve the accuracy of N staging. The positive predictive value, negative predictive value, sensitivity, and specificity of this model were 95.2%, 95.3%, 95.3%, and 95.2%, respectively. The AUC and accuracy were 0.994 and 95.7%, respectively. Following deep learning for metastasis evaluation by MRI examination, the lymph node metastasis rate for rectal cancer patients increased significantly, substantially greater than that for the traditional manual examination method. Similarly, a systematic review (53) showed that radiologists and deep learning models had AUCs of 0.688 (0.603–0.772) and 0.917 (0.882–0.952), respectively. The performance of the deep learning model was better than that of the radiologists, and the artificial intelligence model may more accurately predict the lymph node metastasis of rectal cancer. However, existing studies on the application of deep learning in the diagnosis of rectal cancer lymph node metastasis are sparse, and the findings warrant further investigation.

In recent years, with the development of computer technology, the development of medical imaging analysis methods has been considerably promoted. Methods like conventional radiomics and deep learning have gradually been applied to medical imaging analysis. The accuracy of the results of deep learning and radiomics models to detect lymph node metastasis is shown in **Table 2**.

**Table 2:** Accuracy of results of deep learning and conventional radiomics models to detect lymph node metastasis.

PN/LN	Sensitivity (%)	Specificity (%)	Accuracy (%)	AUROC	95%CI	Reference
Deep Learning						
183	–	–	–	0.920	0.876-0.964	(54)
107	88.9	93.5	91.6	0.912	0.842-0.958	(55)
100	–	–	–	0.8862	–	(56)
414	–	–	–	0.912	–	(57)
619	–	–	94.4	–	–	(58)
Radiomics						
308	50.74	74.42	63.96	0.650	0.583-0.713	(59)
41	85.0	82.0	83.0	0.780	0.630-0.920	(60)
72	–	–	–	0.900	0.800-0.990	(61)
130	82.8	73.3	75.4	0.818	0.731-0.905	(62)
148	73.0	56.6	63.7	0.697	0.612-0.781	(30)
115	–	–	–	0.857	0.726-0.989	(48)
200	–	–	–	0.778	0.769-0.787	(46)
65	–	–	–	0.884	–	(47)
63	–	–	–	0.832	0.717-0.915	(63)
48	–	–	–	0.891	0.799-0.983	(64)
39	80.0	95.8	89.7	0.942	–	(65)
91	89.81	82.57	87.77	0.92	–	(66)
72	94.7	60.4	69.4	0.812	0.703-0.895	(67)
228	89	82	88	–	–	(68)
17/43LN	–	–	–	0.910	–	(69)
220LN	89	82	88	0.855	0.801-0.898	(70)

PN, patient number; LN, lymph nodes; AUROC, area under the receiver operating characteristic curve; CI, confidence interval.

The **Table 3** shows the advantages and disadvantages of traditional imaging versus artificial intelligence. Conventional imaging generally relies on human experience and judgement, whereas artificial intelligence uses predictive algorithms to provide rapid results with high accuracy. Conventional imaging has established costs, while the costs for artificial intelligence systems tend to become more cost-effective over time. Artificial intelligence systems surpass conventional imaging by providing standardized results but may lack the personal touch brought by human interaction.

**Table 3:** Comparing the advantages and disadvantages of traditional imaging and artificial intelligence.

Aspect	Traditional imaging	Artificial intelligence
Advantages		
Speed Interpretation Cost Availability Consistency	Fast initial results Human touch, experience-based judgement Established, standard costs Widely available technology & practitioners Variability due to differing expertise	Predictive algorithms provide rapid results High accuracy, adaptable models Cost-effective in the long-run Increasing availability as technology evolves Standardized results across different systems
Disadvantages		
Speed Interpretation Cost Availability	Limited by human interpretation speed Subject to human error, bias Potential for overuse, unnecessary testing Not always accurate	Potential biases in training data Initial high costs for implementation Limited availability in some areas Lack of interpersonal interaction & touch

## 4 Evaluation of lymph node metastasis in rectal cancer by MDT

Preoperative MDT evaluation is linked to the improvement in the long-term survival rate of patients with locally advanced rectal cancer. Owing to the development in assessing preoperative radioactive tumor staging and neoadjuvant therapy, multidisciplinary group discussions have been introduced. A systematic review study (71) showed that the basis of modern treatment strategies is accurate high-resolution imaging to guide neoadjuvant therapy and precision surgery, followed by detailed pathological examinations to determine important prognostic factors for adjuvant chemotherapy. However, these approaches rely on the close cooperation of interrelated disciplines within an MDT and this multidisciplinary forum is becoming the standard for rectal cancer treatment in the United Kingdom, Europe, and the United States. The fundamental components of modern rectal cancer management are evaluated by the MDT to provide colorectal surgeons with the information they need to guide the patient for the best care. Yu L et al. (72) examined the accuracy of MRI evaluation in diagnosing preoperative staging of rectal cancer by an MDT. The results of the study showed that the accuracy of MRI in diagnosing the N stage of patients by an MDT before surgery was significantly higher than that of the non-MDT group (56.2% vs. 42.1%, P=0.021). For patients without lymph node metastasis, the accuracy of MRI by an MDT was higher (61.2% vs. 37.8%, P=0.009). Thus, MDT assessment improves the accuracy of MRI in the preoperative staging diagnosis of rectal cancer. Based on MDT, the clinical stage of patients can be more identified accurately, which is conducive to choosing better treatment strategies.

## 5 Biomarker-based evaluation of lymph node metastasis in rectal cancer

Biomarkers are molecular models that can be used as a tool for the early detection and personalized treatment of colorectal cancer. These can be classified as diagnostic, prognostic, or predictive. Biomarkers are useful in determining disease progression and recurrence at different stages of the disease, and provide personalized indicators of treatment effects. Quantitative analysis of PVT1 expression in tumors and adjacent normal tissues of 210 patients with colorectal cancer showed that its expression related to tumor differentiation, invasion, high grade, and lymph node spread increased by 51.4% (73). However, not all colorectal cancer cell lines exhibit aggressive behavior which is attributable to the expression of PVT1. The HCT116 colorectal cancer cell line did not show more aggressiveness than the control cell line (74). Gharib et al. studied PVT1 expression as a biomarker of lymph node metastasis but found that when it was used instead as part of a biomarker group including PVT1, HOTTIP, and UCA1, the AUC was higher compared to its expression alone (75). In another study, six EMT-related biomarkers (E-cadherin, N-cadherin, cytoplasmic b-catenin, nuclear b-catenin, Snail, and Twist) and two clinicopathological variables were selected to devise an SVM model, and the sensitivity, specificity and overall accuracy of the model in predicting lymph node metastasis were 68.3%, 81.1%, and 72.3%, respectively (76). Moreover, microRNA−129 (miR-129) is reportedly involved in the metastasis of various malignant tumors, and experimental results show that miR-129 may act as a tumor suppressor in colorectal cancer by inhibiting proliferation, migration, invasion, and EMT of these cells (77). Therefore, future studies can explore the role of miR-129 as a biomarker for the diagnosis and prediction of colorectal cancer. The accuracy of biomarkers to detect lymph node metastasis is shown in **Table 4**. At present, the molecular differences between rectal cancer and colon cancer are gradually becoming apparent, and future research should strictly distinguish the biomarkers for predicting rectal cancer and colon cancer.

**Table 4:** Accuracy of biomarkers in detecting lymph node metastasis.

Biomarkers	Description	Reference
GSN and PRDX4	downregulation of GSN and PRDX4	(78)
CDH1, CDH13, MINT3, CXCL12, RARB, APC	gene methylation	(79)
E-cadherin, N-cadherin, cytoplasmic b-catenin, nuclear b-catenin, Snail and Twist	epithelial-mesenchymal transition (EMT)-related biomarkers	(76)
CD133(+) and CD133(+) CXCR4(+) cancer cells	specific cell surface markers	(80)
miR-19a	upregulation of miR-19a	(81)
miR-129	upregulation of miR-129	(77)
F-cadherin	tumor-related factors	(82)

## 6 Metagenomics evaluation of lymph node metastasis in rectal cancer

In recent years, with the deep exploration of gut microbes, several studies have found that the occurrence and development of colorectal cancer are closely related to the changes in the gut microbiota (83, 84). Advanced metagenomics technologies can detect more potentially pathogenic microbiomes, and metagenome-wide association studies have enabled high-resolution associations between the human microbiome and colorectal cancer (85). The intestinal microbiota can be used as a non-invasive predictive disease biomarker for the occurrence and development of colorectal cancer. Yu (86) performed a metagenome-wide association study on fecal samples from 74 colorectal cancer patients and 54 controls from China and confirmed the association of Fusobacterium nucleatum (Fn) with colorectal cancer in 16 patients and 24 controls from Denmark. Moreover, in the French and Austrian cohorts, four genes were found to distinguish colorectal cancer metagenome from the control group with an AUC of 0.72 and 0.77, respectively, and qPCR testing of two genes, enriched in the early cases, accurately classified colorectal cancer patients in the independent Chinese cohort (AUC=0.84, OR=23). This demonstrates the potential of metagenomics of fecal microbiota as a marker for the diagnosis of early colorectal cancer. In another study, a meta-analysis of 1042 fecal metagenomic samples from seven publicly available studies yielded a better predictor of colorectal cancer based on a functional analysis-based interpretable machine learning approach, which distinguished adenoma samples (87). This approach is promising to prevent colorectal cancer by detecting it in the early stages, making the treatment easier and effective.

Studies on Fn and colorectal cancer show an inseparable relationship with the latter’s occurrence and development, including the research from European molecular biology laboratories on colorectal cancer specimens from eight geographically different regions using fecal shotgun metagenomics meta-analysis. Fn in colorectal cancer metagenomes (false discovery rate (FDR) <1 × 10 ^ (–5)) provides important clues for a better understanding of the relationship between the two (88). Another study, which analyzed 969 stool samples from colorectal cancer patients and healthy controls using metagenomic sequencing analysis, showed that the intestinal tracts of colorectal cancer patients had abundant Fn and the composition of these bacteria was characterized for different data sets, even without model training. It maintained high accuracy (AUC=0.84) (89). Castellin et al. (90) performed metagenomic analysis to compare the genomic DNA of colorectal cancer and normal tissues, and found that the number of Fn in the former group was 415 times higher compared to the surrounding tissues. Therefore, we boldly speculate that the high probability of the occurrence and development of colorectal cancer is related to the enrichment of Fn. More studies and data are needed to support these results, and greater challenges for future in-depth research remain.

Lymph node metastasis and distant metastasis usually occur with the progression of colorectal cancer. Yan et al. (91) have discussed the role of Fn in colorectal cancer metastasis. In a retrospective cohort study, they found that the feces of patients with metastatic colorectal cancer contained a large number of Fn. There was a strong association between the number of bacteria and tumor invasion, lymph node metastasis, and distant metastasis. In another study, Mauro Castellarin (92) performed RNA-sequencing to screen colorectal cancer and match normal tissue samples. The host sequence was subtracted. Fn sequences were significantly overexpressed in tumor samples relative to the controls. qPCR analysis of 99 subjects validated excess Fusobacterium sequences in tumors versus matched normal control tissues (p = 2.5 × 10 ^ (–6)). A significant positive association was found between Fusobacterium and lymph node metastasis. With the development of fecal microbial metagenomic analysis techniques, major breakthroughs have been made in the prediction of lymph node metastasis of colorectal cancer. Although relatively few studies have been conducted, this is a new opportunity to study whether bacteria such as the abundance of Fn can accurately diagnose colorectal cancer, bringing more possibilities for future scientific research.

## 7 Challenges and future perspectives

While many advances in diagnostic techniques are encouraging and impressive, considerable challenges remain in their application for detecting lymph node metastases in rectal cancer. Accurate diagnosis of lymph node metastasis in rectal cancer requires the use of bioomics technology or imaging to collect data based on computer-based biological analysis, and computational biological analysis needs to be driven by medical data. Therefore, data standardization and model generalization are key factors for the success or failure of the diagnosis of lymph node metastasis in rectal cancer. We discuss these in the following subsections.

### 7.1 Data standardization

At present, there is a general lack of high-quality data for standardization. The existing data sets have various standards, large systematic deviations, and lack unified understanding of diseases. The lack of a unified and clear standardized description of data leads to interaction barriers between machine learning and actual data, and the machine misunderstands the true meaning of data. Therefore, it is urgent to solve the technical problem of medical data standardization. Establishing a standardized database can improve the quality of medical data, maximize the value of scientific data, assist the construction of models, and apply them to the field of clinical diagnosis of diseases (93).

### 7.2 Model generalization

The generalization of models is critical to their clinical application (94). Simply put, the generalization of the model is mainly reflected in its reproducibility (95), indicative of the performance of the prediction model for similarly-distributed data. However, the actual situation is that most models have excellent performance in training data but do show stable performance in internal and external independent verification, that is, the model’s generalization is poor. There are many possible reasons but the main one is the small sample size of the data. Therefore, the generalization of the model can be improved by increasing the sample size of the data. Therefore, multi-center research is the future direction in the field. By combining multi-center data, the problem of sample size can be solved, the reproducibility can be improved, and the generalization of the model can be enhanced.

## 8 Conclusion

Although conventional imaging methods for rectal cancer have certain limitations for judging lymph node metastasis in rectal cancer, medical imaging analysis based on computer technology has achieved unprecedented development in recent years. Moreover, its ability for image recognition and natural language extraction has undergone continuous improvement and is expected to play an increasingly important role in the diagnosis and treatment of rectal cancer. The MDT model combines opinions from multidisciplinary experts to provide more accurate clinical information and guide better clinical decision-making. Biomarkers are useful for determining disease progression and recurrence at various stages of the disease, and provide personalized indicators of treatment efficacy, thereby improving clinical diagnosis accuracy, and providing better and more powerful information for planning treatment. Metagenomic studies of fecal microbes may improve the accuracy of predicting early rectal cancer and, in the future, may predict lymph node metastasis in rectal cancer at the microbe level. In recent years, owing to the rapid development of imaging and omics technologies, researchers have used high-throughput methods and computer techniques to mine a large amount of information from these data. We believe that by integrating artificial intelligence technology and imaging or omics data to build an efficient and generalized disease diagnosis model, accurate diagnosis and evaluation of lymph node metastasis in rectal cancer is possible, along with the provision of better treatment for patients, and ultimately help improve patients’ prognoses and quality of life. With the joint efforts of data scientists and clinicians at home and abroad, the development prospect of diagnosis of lymph node metastasis in rectal cancer has bright prospects.

## Author contributions

WP, writing—original draft. YG, project administration, revised, and edited manuscript. HQ and LM, supervision. All authors contributed to the article and approved the submitted version.
